# Measuring the Physical Activity of Seniors before and during COVID-19 Restrictions in the Czech Republic

**DOI:** 10.3390/healthcare10030460

**Published:** 2022-03-01

**Authors:** Vít Janovský, Marek Piorecký, Jan Včelák, Michael Mrissa

**Affiliations:** 1 University Centre for Energy Efficient Buildings, Czech Technical University in Prague, 273 43 Buštěhrad, Czech Republic; jan.vcelak@cvut.cz; 2 Faculty of Biomedical Engineering, Czech Technical University in Prague, 272 01 Kladno, Czech Republic; marek.piorecky@fbmi.cvut.cz; 3 National Institute of Mental Health, 250 67 Klecany, Czech Republic; 4 InnoRenew CoE, Livade 6, 6310 Izola, Slovenia; michael.mrissa@innorenew.eu; 5 Faculty of Mathematics, Natural Sciences and Information Technology, University of Primorska, 6000 Koper, Slovenia

**Keywords:** pandemic, physical health, motion tracker

## Abstract

Social workers require a better understanding of the impact of pandemic measures on the level of physical activity of their clients to better target client activation. In this retrospective tracker-based study (two years of measurement), we examined changes in the physical activity of the elderly population (204 participants with an average age of 84.5 years) in the Czech Republic as a result of measures to prevent the spread of COVID-19. Physical activity was statistically compared according to the physical, demographic and social conditions of the participants. In addition to observing the expected activity decrease during the COVID-19 pandemic, we made several hypotheses based on the sex, age group, body mass index, type of housing (apartment or house) and size of the city of residence. We found that 33% of the 204 participants had increased levels of physical activity in the period following the COVID-19 pandemic outbreak in Central Europe. We found that the size of the city where the seniors lived and the type of housing did not affect the general level of physical activity. When comparing physical acquisition rates in each month of 2019 and 2020, we saw the largest declines in April and May 2020, that is, one month after the start of the lockdown.

## 1. Introduction

Necessary restrictions during the COVID-19 pandemic forced social isolation of the population, and this led to a reduction in physical activity. (PA) [1]. Reduced PA is due to reduced community activities (shopping, visiting a library, pub or coffee) and reducing formal exercise (day-care centre, walks or trips). A reduction in physical activity level (PAL) has adverse health consequences for the elderly [2]. The aging process, regardless of disease, is accompanied by many changes in the body systems of the human organism.

Changes occur in both the structure and function of organs. Many of these changes are characterised by a decline in physiological reserves to such an extent that, even when basic functions are preserved, organ systems become progressively less able to maintain homeostasis and to cope with the rigours of the environment, disease and medical therapy. These changes can cause increased vulnerability of the body, a decline in certain abilities and a related decline in overall performance. It is important to distinguish the changes associated with natural ageing from pathological processes and to plan interventions appropriately. A decline in PA leads to degradation of the body [3,4]. The elderly population is one of the most severely impacted groups [5] and is the focus of our study.

Several research works have looked at how COVID-19 measures impact PAL in different countries. However, most of the research is based on questionnaire data [6,7,8,9,10,11]. International online survey research with 1047 responses revealed that daily sitting time increased from five to eight hours per day and had a negative effect on all levels of intensity of PA (vigorous, moderate, walking and overall) [2]. 

The same results were obtained with questionnaires [12] in Dutch patients with cardiovascular disease. Sedentary behaviour increased by 55 (−72, 186) minutes per day [median (interquartile range)]. A review [13] of peer-reviewed articles identified the greatest threats are to the elderly. They face social isolation, which affects both their physical and mental health. Moreover, they also suffer economic hardship. Psychological well-being can have a big impact on subjective evaluation [14]. The negative influence of COVID-19 restrictions affects the mood of a person and responses to the questionnaire. Research also shows that low PA causes damaging psychological effects [8].

In a study of the risks of increased inactivity during the COVID-19 outbreak, the authors highlight the need for research on PA during the COVID-19 pandemic based on objective assessment tools (accelerometers and pedometers) [15]. Their research question is about defining the actual impact of social isolation on PAL. A study based on tracker data focusing on activity reduction due to COVID-19 measures for the elderly (around 80 to 90 years old and older) is still missing.

Therefore, the impact of these COVID-19 restrictions on the PAL of seniors at home remains unexplored. Hence, it is also important to keep in mind that inactivity is the fourth leading cause of mortality according to the World Health Organization [16]. It is necessary to address the non-subjective way of assessing the impact of COVID-19 restrictions on the PA of the elderly. Our research work aims to determine whether there was indeed a decline in PA during the COVID-19 pandemic, and if so, to identify the most influential factors. We also evaluated the relationship between the results of the subjective questionnaire survey and the objective measurement of the change in PAL.

## 2. Materials and Methods

### 2.1. Participants

Seniors (participants) are clients of social services (Emergency Care), live at home and use a daily GPS tracker [17] with motion detection. The data on research participants are anonymised and do not contain any personally identifiable information about the proband. Researchers were only given the data necessary to evaluate the research. A full list is provided in Appendix A. Data were exported from the Emergency Care system in June 2021. 

**Figure 1:**
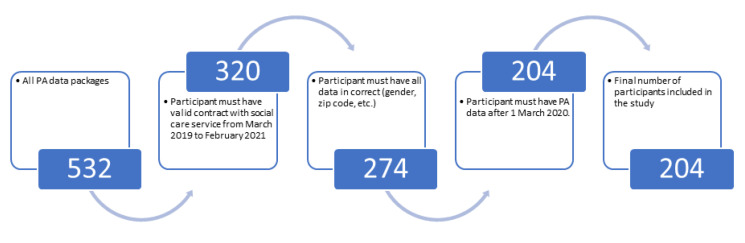
The process of filtering participants leading to the final dataset.

This social service operates throughout the Czech Republic, and the selection of participants is random. For our research, we received data on 500 clients. We performed a manual check of the data according to the criteria in Figure 1, and non-compliant probands were excluded from the study. Participants who met the following criteria were included in the retrospective study and were social service clients between March 2019 and February 2021 who had correctly completed data (gender, zip code etc.).

#### 2.1.1. Data Filtering

Sometimes participants do not use their tracker devices. This happens due to hospitalization, holidays with family or other reasons. That does not mean that they were not moving at the time. Therefore, we excluded from the statistics days when clients had no record of activity (days with zero PA were not counted). If the period when the participants did not have a PA meter active was over the critical date (1 March 2020), they were also excluded from the statistics to maintain the same limit and the first reaction to the introduction of the restriction. We found an average record of one proband for 351 days out of 365 days in a year. In total, the valid data contains 143,515 days of records over the entire two-year period. After filtering the participants and data, as shown in Figure 1, we obtained 204 valid participants. 

#### 2.1.2. Participant Statistics

All participants wore a GPS tracker BlueIdea watch version 01 (Producer Takit, Hong Kong) for two years. This tracker takes the form of a watch and uses the GSM network for communications. The device uses a pedometer (Freescale MMA9555) and a G sensor (MOSCH BMA223) to record PA.

For statistics on the size of the cities in which participants live, we use public information about ZIP codes and city names [18] on the Czech post website and the statistics document from the Czech statistics government website [19]. We only had information about the ZIP code of participants. In the first step, we found the name of the city from the Czech post document by ZIP code. In the second step, we found the number of residents in the Czech statistics government document by the name of the city. In the last step, we compared the number of residents for each participant.

Participants with a “with help” mobility status use a cane, or another means of help to move. In this study, there were two participants with a wheelchair, which is less than 1%. All information about the participants is summarized in Table 1.

### 2.2. Data Package

This article shows the change in PAL in the elderly population based on real data from personal trackers. This device uses an accelerometer for PA classification. We used aggregated PA data at regular ten-minute intervals. We used several data packages. The structure and content of the data packages is described in Appendix A. PA (see Appendix A.1) and characteristic data (see Appendix A.2) are from the emergency social care system. This participant data was anonymised and does not contain personal data. The year of reference includes one year of data before March 2020 and one year after. The data are in the JSON format. Information about the city population (see Appendix A.3) [19] and ZIP codes (see Appendix A.4) were obtained from a public website [18]. 

Social services have adopted the following methods to encourage people to wear devices:

- The system has information about the battery; it knows when the device is put on the charger and when it is removed from it.
- The system monitors a “no motion alarm” in individual wear time (usually 7 am to 10 pm). If a device does not detect motion in a two-hour time window, then the caregiver will contact the user.
- The system monitors “long charging” in individual time (usually up to 8 am). If the battery device is over 98% and without motion, then the operator will contact the user.
- The system monitors “low battery” and “lost data”. Again, the caregiver will contact the user.
- There is regular training on the use of the equipment.

When processing data, we assume that clients always carry the device with them during the active day. They do not wear it only when they sleep. Therefore, we only count on PA data between 7 am and 10 pm.

### 2.3. Statistical Analysis

Data were processed in the MATLAB2017b (The MathWorks, Inc., Natick, MA, USA) software environment. Hypotheses H01 to H12 regarding participant activity for different conditions (gender, age etc.) were statistically tested to conditions of a parametric/nonparametric test. The Lilliefors test was used to evaluate the distribution of normality. This test does not have to specify the expected value and variance of the distribution in the null hypothesis. With respect to the log–normal distribution, the data without subconditions (complete dataset) were logarithmized to use the parametric paired t-test (correspond to H01). For subgroups that did not contain such a large amount of data, a nonparametric variant of the Wilcoxon test was used.

Each tested group is described by descriptive parameters: the mean, standard deviation, median and interquartile range. The mean and standard deviation are parameters that characterize data with a normal probability distribution. The median corresponds to the mean and interquartile range of the standard deviation for data that do not have a normal probability distribution. *p*-values reflecting the result of testing the null hypothesis are the output of the t-test or Wilcoxon paired/unpaired test. *p*-values of normality distribution tests are also given here. All statistical values are given in Appendix B.

For the entire dataset (men and women together without other exclusion conditions), the data were divided into two paired groups: before and after the COVID period. A normality test was performed, in the case of a paired *t*-test from group X, with the proviso that the pairing data is automatically assumed to meet the normality X’, which corresponds to the second, paired group. The *p*-value of the Lilliefors normality test was 0.005. The size of both selections was 204 for each group. Cohen’s D is 0.286. In this case, the type II error (beta) is about 55%, and the strength is 45%. In the theoretical case of necessity 80% power (beta 20%) for the same mean values, it would be necessary to have a selection size of 568.

### 2.4. Established Hypotheses

For the evaluation, we set the following hypotheses. Table A1 contains *p*-values for these hypotheses.

- Hypothesis 1 (H01). Participant PA in the pre-COVID period is the same as in the during-COVID period in general.
- Hypothesis 2 (H02). Male PA in the pre-COVID period is the same as in the during-COVID period in general.
- Hypothesis 3 (H03). Female PA in the pre-COVID period is the same as in the during-COVID period in general.
- Hypothesis 4 (H04). Participant physical activity in the pre-COVID period is the same as in the during-COVID period for every month.
- Hypothesis 5 (H05). The PA is the same for pre-COVID and during-COVID periods in a big city.
- Hypothesis 6 (H06). The PA of participants living in a small or big city is the same in the pre-COVID period.
- Hypothesis 7 (H07). The PA of participants living in a small or big city is the same in the during-COVID period.
- Hypothesis 8 (H08). For a participant living in a flat, is PA the same for a participant living in a house in the pre-COVID period.
- Hypothesis 9 (H09). For a participant living in a flat, is PA the same for a participant living in a house in the during-COVID period.
- Hypothesis 10 (H10). Physical activity in the pre-COVID and during-COVID periods is the same for a given age category.
- Hypothesis 11 (H11). BMI is correlated with average PA in the pre-COVID period.
- Hypothesis 12 (H12). BMI is correlated with average PA in the during-COVID period.

## 3. Results

Our research work aims to find out whether there was indeed a decline in PA during the COVID-19 pandemic, and if so, to identify the most influential factors.

### 3.1. Physical Activity in General

#### 3.1.1. Physical Activity Evaluation

First, we compared PA over the periods and compared our findings with questionnaire-based research. We compared the pre-COVID PA data (1 March 2019 to 29 February 2020) with the during-COVID data (1 March 2020 to 28 February 2021) in general. We set up the H01, H02 and H03 hypotheses. There was a decrease in PA among the elderly in the during-COVID period compared to the pre-COVID period, as we expected. There were no significant differences between the female and male groups. Women had a pre-COVID average of 14% of active time and men 9.6%. In the during-COVID period, women’s active time fell to 12.5% and men’s to 8.4%. A value of 100% activity time corresponds to PA in every minute of the active part of the day (from 7 am to 10 pm). For women, active time fell from 126 min per day to 112.5 min.

#### 3.1.2. Physical Activity Calculation of the Rate of Decline

**Figure 2:**
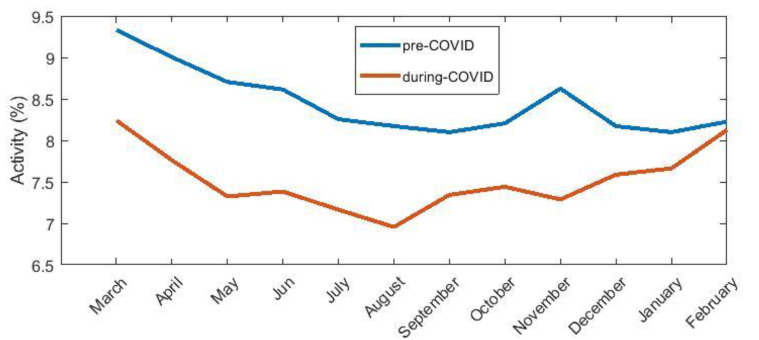
Graph showing activity of all groups before and during the COVID-19 pandemic. The *x*-axis represents time information with a step of one month; the *y*-axis represents the activity value. A value of 100% activity corresponds to PA in every minute of the active part of the day (from 7 am to 10 pm). The pre-COVID period is shown in blue; the during-COVID period is shown in red.

We computed the PA data for 204 participants. We had 143,515 days of records in two years. Thus, we had, on average, over 351 days of records for one participant in one period. If we compare the pre-COVID PA data with the during-COVID, overall, PA decreased by 12.3%. In the women’s group, PA decreased by 12%. In the men’s group, PA decreased by 14%. Plots of PA over the study period are shown in Figure 2. Statistical evaluations for all months is presented in Table A2. 

Overall, the activity before COVID was higher (mean value = 8.894) than after COVID (mean value = 7.885). A total of 58% of participants decreased their PA level between pre-COVID and during-COVID periods, compared to 42% increased their PA. An overview of the values is given in Table 2.

**Figure 3:**
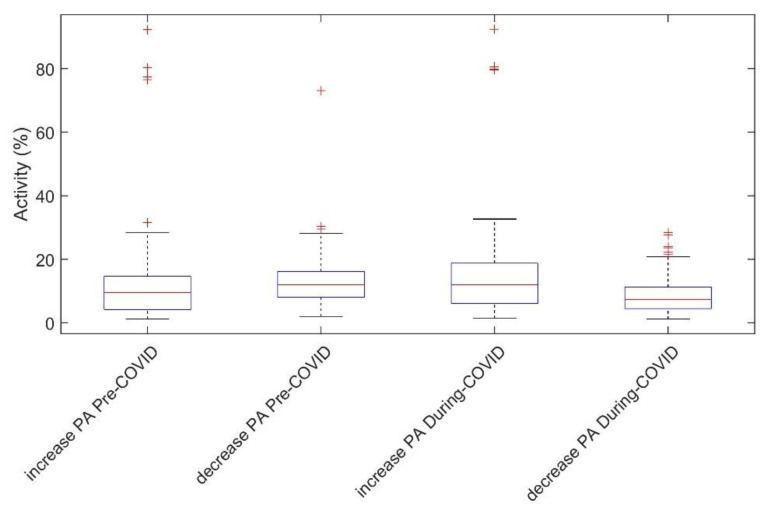
Box plot plots representing groups of individuals for whom an increase and decrease in activity were observed before and after COVID-19-related restrictions. Red crosses represent outliers.

Overall, those people who had reduced activity during COVID-19 had slightly more activity before restrictions (opposite the other group) and also had a thinner interquartile range; see Figure 3. The third box (the during-COVID group with increasing PA) had a wider box. This can be interpreted as meaning that the increase in activity was “unhomogenic”, while the fourth box shows that the decrease in activity appears relatively homogeneous, and there are not so many outliers (red crosses—calculated by Tukey’s method).

Thus, we find it difficult to identify a parameter that quantifies the PA of the group where the activity increased because it does not behave as homogeneously as the group where the decrease occurred.

### 3.2. Physical Activity in Each Month

COVID-19 measures continued throughout the year. We were interested to investigate whether there was a demonstrable decline in PAL in all months. We compared the pre-COVID PA data with the during-COVID data for every month. By comparing data from two identical months—for example, January 2020 with data from January 2021—we do not need to account for the aspect of changes in PA due to cold weather. Another aspect that may influence the results is the fact that people are a year older. 

This can negatively affect their physical fitness. We set up the H04 hypothesis. Only April, May, June and November had a significant *p*-value for our hypothesis. These months have seen a significant change in PAL. For multiple comparisons, we applied a correction. Using the Bonferroni correction, there are statistically significant changes between activity before and during the COVID-19 pandemic at four months. Using a milder FDR correction, a statistically significant change was also observable in August.

The trend in PA levels throughout the follow-up period is shown in Figure 2. We look deeper into the two most statistically different months. The PA of the participants in April and May had the most statistically different values. For April, there was a 21% decrease in PA for men and a 13% decrease for women. For May, there was a 36% decrease in PA for men and a 17% decrease for women. A comparison of the average PA between men and women is shown in Table 3.

### 3.3. Physical Activity in Different City Size

Government measures were nationwide in scope. We wondered, however, whether they had the same impact on people living in big cities and outside them. We compared the pre-COVID PA data with the during-COVID data in different sizes of cities. We define a city as a big city if it has more than 10,000 inhabitants and a city as a small city if it has less than 10,000 inhabitants. 

We set up the H05, H06 and H07 hypotheses. The *p*-value was calculated by the paired *t*-test for all hypotheses. We computed PA data for 204 participants that had information about their city. If we compare the big city PA data with the small city data in the pre-COVID and during-COVID periods, the PA variation was the same. In the big city, PAL declined from 11.4% of the active part of the day to 10.3%. In the small city, the decline was from 15% to 13.1%. Therefore, we can conclude that the size of the city did not impact the change of PAL.

### 3.4. Physical Activity in Different Households

Most people living in a house have a private garden to roam. Is this an aspect that was reflected in the PAL? We compared the pre-COVID PA data with the during-COVID data in different households. We set up the H08 and H09 hypotheses. We computed PA data for 191 participants that had information about the type of living. Comparing the PA of flat and house living participants, we did not find any significant changes. PAL for both groups are shown in Table A3.

### 3.5. Physical Activity in Different Age Groups

For the social worker activation program, it is important to know which age group is most affected by the restriction and to focus on it. We compared the pre-COVID PA data with the during-COVID data in different age groups. We set up four similar large age groups. We set up the H10 hypothesis. An interesting finding is that the second age group (80 to 85 years) was the most dominant. The specific *p*-values for each group are in Table 4.

Based on these findings, we can say that there was a statistically significant difference in the pre-COVID and during-COVID periods only in the 80–85 years age group after Bonferroni correction (for FDR correction, this was also statistically significant in the 85–90 age group). The most active age group was 86–90 years. This group had an average of 15.2% of active time in the pre-COVID period. In the during-COVID period, the value declined to 14.6%. This is still more than other age groups in the pre-COVID period. See Table A3 for detailed results.

### 3.6. Correlation of BMI (Body Mass Index) with PAL

We wondered if BMI influenced PAL in our group. We investigated the correlation of BMI with PA level. For this research, we defined the H11 and H12 hypotheses. The correlation values are in Table A4. We can say that there was no statistically significant correlation of BMI with PA level in the pre-COVID or during-COVID periods.

### 3.7. Variance of Physical Activity Values

Some participants had a large variance in PA values. The variance of PA values was quite large. If we look at the variance over months (average activity over months for each participant), it was large as well. Thus, some had relatively stable PA values throughout the year, and some had large fluctuations. We describe our explanation of this phenomenon in the discussion.

## 4. Discussion

The aim of the study was to verify the results of subjective questionnaire surveys on the impact of COVID-19 measures on the PA of the elderly population in Central Europe. Our results, based on objective measures of PA, showed a consistent overall decline in PAL. However, the absolute decrease was considerably smaller. 

We combined PA data in pre-COVID and during-COVID periods with information about the demographics to estimate the effects of disease prevention and government restriction orders on the level of PA. There is little research using a device-based measure of PA related to COVID-19, although we found some [20,21,22,23]. These four works were not comparable to our article (two-year period, average age 84.5 and 204 participants). 

The Italian work [20] had a study period of only one week. The study from Spain [21] had only 20 young people with a mean age of 22.6. The study with cardiac patients [22] had only 26 participants with an average age of 58.8 years and a period of six weeks. The decrease of PAL was 16.2% in this group.

Only one paper had information about the age group of 65+ years [24]. In that study, the 65+ year age group had increasing PAL. In the research, smartphones were used as tracking devices. However, the study focused on all age groups, and therefore the 65+ year group was not examined in detail. The average age of the participants in that study was 41 years. Most seniors over the age of 80 do not have a smartphone, and thus data in the age category 65+ [24] for that study did not include all people in this category as the research was limited to smartphone users only.

Comparing with other studies is difficult because we are comparing two periods of one year each. Questionnaire-based research usually compares PA in months or only generally in pre-, during- and post-COVID periods. Tracker-based research usually compares PA in a one-week period because they have data only from a few months. However, in general, our observation of the lower elderly (204 participant groups with an average age of 84.55 years) PA during the first year of the COVID-19 situation is in line with other research [6,12,25,26]. Our long-term (two years) study showed decreases in PA by comparing entire periods. The largest decline in PA was among men at 14%. In the during-COVID period, the overall participant PA was 12.3% lower than in the pre-COVID period. These decreased values are considerably smaller than the findings of the questionnaire surveys [2,27]. 

In one study [27], statistical results provided insight into PA patterns before and during isolation. The number of hours per week decreased by 30.27% for the 65+ age group during the lockdown. This questionnaire-based survey reported a 33.5% decrease in total PA in minutes per day [2]. This may be due to the large difference in participants. In this study, participants were mainly from North Africa and Western Asia, and only 9.8% of participants were over 55 years of age. The elderly in our age group likely do not engage in sufficient exercise outdoors in general. 

Therefore, their limitations in outdoor exercise did not have a great impact on the overall PA. This explains why the drop in overall PAL may be so much lower than the rest of the population. We have the same explanation for the different values of the decline in PA for the tracker-based research. In a FitBit study [28] based on data from FitBit trackers, Czech users had a 20% decrease in the number of steps in the week ending 22 March 2020. FitBit products are typically used by users who are younger than our participant group. Therefore, we assume a different average age of participants.

The same problem as when comparing our results is the case with our comparison of PA over months. All studies compared PA data between consecutive months. In contrast, we compared the decline in PA between the same month last year. Changing seasons and weather changes have a large impact on the overall PA of the elderly. Research in the UK [6] supports this claim. All age groups had a decrease in PA when comparing the two periods (January 2020 to 18 March 2020) and (18 March 2020 to June 2020), except the 65+ age group. In contrast, this age group was the only one to experience an increase in PA. 

Some participants in our study also reported an overall increase in PA. Thus, our results show that an increase in PA is possible in this age group. In winter, the elderly show less outdoor and overall activity. On the other hand, comparing PA in a month in 2019 versus the same month in 2020 may be affected by the manifestations of old age and changes in the participant’s condition over a year. However, the decline in PAL was only evident in some months; thus, the effect of ageing likely did not affect the results.

The results of our research on the correlation between BMI and PAL (pre-COVID period r = 0.004 and during-COVID period r = 0.121) had the same conclusion as the research from this year. The Urzeala questionnaire research [27] showed an almost zero correlation between BMI and PA in before-COVID time (r = −0.08) and during-COVID time (r = 0.07). BMI and PAL do not correlate with each other, which may mean that BMI does not negatively affect PAL rates. Being overweight is not a barrier to PA.

Our research comparing PA rates in the pre-COVID period with PA rates in the during-COVID period showed that, overall, PA decreased in the senior group (dataset with 204 participants, average age 84.55 years). This objectively confirmed previous research based on subjective data from questionnaires. However, there was a group of 86 participants (42%) that showed an increase in PA despite all COVID-19 pandemic measures. This phenomenon was also described in the study in the UK [24] that used smartphones as trackers. 

The 65+ age group of participants was the only one to experience an increase in PA despite the lockdown period. Isolation and closure of home care services, such as lunch delivery and shopping assistance, may have caused this phenomenon. This left the seniors to fend for themselves and to walk on their own to places of interest. We were unable to find parameters and statistically confirm such hypotheses that would identify key parameters that distinguish this group from other participants. 

We examined the effect of housing (city size and apartment vs. house) and other characteristics, such as BMI, age etc., on the rate of decline in PA. However, none of these aspects played a major role. Gender did not prove to be decisive, either, as the ratio of men to women remained the same in this “rising” group (21:65) as in the whole dataset. Hence, it is necessary to further focus only on the group showing an increase in physical activity, extend this dataset and subject it to detailed examination. In doing so, it could be possible to identify the key conditions stimulating higher PA.

These findings show that research based on non-subjective PA measurement (tracker-based measurement) has a much more detailed and greater predictive value. It is essential to validate conclusions based on questionnaire surveys with accurate measurements where possible.

Overall, PA declined in 118 research participants. This group had the same median age as the group with unchanged and greater PA. Thus, this suggests that the age factor is not that important; it may be, for example, about their state of mind or genetic predispositions. Even the unproven difference in PAL between those living in an apartment and a house shows that living conditions do not have such an impact on PAL.

The two most statistically different months for the PA level were April and May, which correspond to the start of COVID-19 restrictions in the Czech Republic. We had disturbing news about the COVID-19 situation in the world before the start of 2020. In the Czech Republic, the first three cases were confirmed on Sunday, 1 March 2020 [29]. The Czech Republic’s government declared a coronavirus outbreak in its territory due to the emergency health threat (referred to as SARS-CoV-2) on 12 March 2020 [30]. 

Two days later, the government banned retail sales and services (except when necessary). The ban on the free movement of persons was issued on 15 March 2021. All the population had to wear a face mask; many families were scared and isolated their elderly. The situation and the regulations changed daily. Therefore, in future work, we want to focus on a detailed analysis of a larger dataset of the PA in days. There were also special opening hours for shops for the elderly. These specific restrictions could have some impact on the elderly’s PA in our country.

We have the same explanation for our next research result. It has been shown that the size of the city in which a participant lives did not affect the level of PA in general. For the pre-COVID and during-COVID periods, the PA levels follow the same patterns for both groups.

As we have already seen above, PA has its own peculiarities for the elderly. For example, it is not so much influenced by living conditions. Our research did not find a demonstrable difference in the decline in PA between the group living in a house and the group living in an apartment. The older population likely spends most of their time at home, and the ability to use the garden around the house had no demonstrable positive effect on PA levels. Alternatively, the PA could be increased for people living in an apartment through outdoor activities (e.g., shopping). Apartments frequently occur in big cities. We cannot say that people do not go outside in their gardens or visit outdoor activities in the city; however, we did not find an effect on PA levels.

The decline in PA was most statistically demonstrated in the age group 80–85 years, see Table A1. The dominant group was the group between 86 and 90 years of age, after milder FDR correction. We did not find an association between mobility status (a condition that describes how able a participant is to move) and these two groups. The results in Table 5 show the distribution of mobility status for different age groups. The largest proportion of participants had no problems or only small problems.

Research participants in the age group of 80 to 85 years are in a health condition that allows them to have the most PA of all. Younger participants are likely to have had an injury or illness that prevents them from being active. Older categories have greater signs of ageing that also do not allow them as much PA. Therefore, the latter age group had the most demonstrable decline in PA. It is this group that was the most at risk for pandemic restriction, and it is essential to focus on methods to encourage PA despite lockdown. 

It is not only this group that is at increased risk of serious health complications when PA declines. A study [31] using a self-report method to assess physical fitness highlighted that less physically fit patients (63 ± 14.8 years) had a greater risk of major complications, including cardiovascular and neurological events. Regular aerobic exercise in the elderly has a demonstrable effect on blood flow and brain functionality—cognitive fitness [32]. 

As PA declines, seniors are at risk for cognitive decline. Of our proband group, 58% were also at risk of cognitive decline due to the avoidance of regular PA. Social services using personal devices that measure PALs could identify users at risk through long-term assessment of PAL and alert them to the health risks associated with low PA, such as diverticulitis [33]. It is essential to address methods of motivating regular activity during periods of isolation.

In the graphs (Figure 2 and Figure 3) showing the level of PA throughout the study period, it can be seen that, from March 2020, there was a decrease in PA compared to 2019. This could be due to the start of restrictions in the Czech Republic (restrictions on the free movement of persons), which began in March 2020. This conjecture is confirmed by comparing the months of February. The month of February 2020 had activity comparable to February 2019. 

Comparing the trend of PA (Figure 2) in the pre-COVID and during-COVID periods, they are similar. The difference is in the overall level of PA in the during-COVID period. This also confirms the result of hypothesis H01. It can also be seen that there was a significant decrease in PA in November 2020 compared to 2021. This may be because the second wave of the epidemic came in the fall of 2020, and the measures were tightened again. Therefore, all stores closed and reopened in December.

Some participants had a large variance in PA values. This may be due to the absence of PA data on some days. However, we do not include these days in the calculations, i.e., the average is calculated only from the number of days with non-zero PA. Given this fact, this should not affect the variance. We attribute the large variance in values to the irregular PA of the participants. Seniors often spend some days only at home and do not go out. On other days, they go to the store or the doctor. For these cases, the difference between total daily PA is several times higher. Therefore, it is essential to find methods to motivate seniors to perform regular PA at home. PA must be maintained at a minimum level every day. Irregular PA in the elderly increases the risk of injury and health complications.

As our next future work, we consider it appropriate to further study our data to find the common characteristics that identify the most vulnerable group of seniors. For this group, we will then suggest specific measures to reduce the impact of COVID-19 restrictions based on these characteristics. We hope that the findings of the “rising PA” group will help us to do this. Aubertin-Leheudre and Rollandin [34] recommended a set of exercises for older people. These simple, adapted, specific daily physical activities that include strength, balance and walking exercises can be considered the best solution to care for frail older adults during the COVID-19 pandemic. Our task will be to adapt these exercises to the target group and choose the appropriate communication channel.

## 5. Conclusions

Our research comparing PA rates in the pre-COVID period with PA rates in the during-COVID period found that the overall PA declined in the elderly. This objective confirms the research based on subjective questionnaire data. The main difference is in the value of decline, as our value is considerably lower than the results obtained from the questionnaire surveys. We suggest that the elderly in our age group do not engage in sufficient exercise outdoors in general; therefore, their limitations in outdoor exercise due to COVID only had a limited impact on the overall decrease in PA. The most significant decrease in PA was proven to be the one that happened immediately after the first restriction (March 2020). Noticeably, our study also revealed a group of 86 (42%) participants that showed an increase in PA despite COVID-19 restrictions, a phenomenon that has also been described in the literature [11].

Our findings confirm the hypothesis that it is possible to support the PA of elderly during lockdown. Therefore, future work includes studying the factors that influenced the group of participants that showed an increase in PA, with the help of social workers who can help suggest a more focused activation program. We believe that the development of appropriate tools to support the PA of elderly during an upcoming COVID-19 restriction can contribute to a large reduction of PA decline and, therefore, to an improvement of the quality of life.

## Figures and Tables

**Table 1:** Demographic data about the 204 participants.

Group		Number	*n* (%)
Gender	Male	46	23
	Female	158	77
Body	Weight ± SD	79.54 ± 51.7 kg	
	Height ± SD	175.50 ± 8 cm	
	BMI ± SD	25.80 ± 21.2	
Population	Weight in Big city ± SD	81.10 ± 68.3 kg	
	Weight in Small city ± SD	72.33 ± 12.9 kg	
	Age in Big city ± SD	84.26 ± 7.4	
	Age in Small city ± SD	84.31 ± 8.2	
Male	Weight ± SD	79.54 ± 11.1 kg	
	Height ± SD	175.50 ± 6.4 cm	
	BMI ± SD	25.80 ± 3.2	
	Age ± SD	84.80 ± 9.3	
Female	Weight ± SD	76.50 ± 58.4 kg	
	Height ± SD	161.94 ± 5.5 cm	
	BMI ± SD	29.23 ± 24	
	Age ± SD	84.22 ± 7.3	
Mobility	No problems	55	27
	Small problems	80	39
	With help	43	21
	Wheelchair	2	1
	Not defined	24	12
House	In house	43	21
	In flat	98	48
	With family	39	19
	Not defined	24	12
City size	Over than 100 k. (Big city)	113	55
	Less than 100 k. (Small city)	91	45
Age group	<80	44	22
	80–85	49	24
	86–90	58	28
	>90	53	26
Age	Average age	84.55	
	Max age	101	
	Min age	57	

**Table 2:** Number of participants with decreased, unchanged or increased PA when comparing pre-COVID and during-COVID periods.

Status	Number	(Percentage)	Age ± SD	PA Difference
PA has declined	118	58%	85.11 ± 7.37	5.49%
PA has increased	86	42%	83.98 ± 8.17	2.03%

**Table 3:** The most statistically significant months when comparing physical activity in the pre-COVID and during-COVID periods and comparing the average activity between male (M) and female (F).

Month	Pre-COVID		During-COVID	
	M	F	M	F
April	9.88	14.56	7.78	12.61
May	10.69	13.98	6.87	11.58

**Table 4:** Comparison of pre-COVID and during-COVID PA for different age groups.

Age Group (Years)	*p*-Value
<80	0.471 (α = 0.013)
80–85	0.001 (α = 0.013)
86–90	0.034 (α = 0.013)
>90	0.234 (α = 0.013)

**Table 5:** The distribution of mobility status for different age groups.

Age Group (Years)	No Problems	Small Problems	With Help	Wheelchair	Not Defined
<80	18	12	6	1	7
80–85	15	38	9	1	2
86–90	13	20	13	0	11
>90	9	10	15	0	4

## Data Availability

Physical activity and client data are available upon request. To request data, readers should contact the corresponding author: V.J. Other input data are public, and the reader can use a link in the references section. Details regarding the analytical methods including MATLAB scripts can be requested from the corresponding author: M.P. The data are not publicly available due to big size.

## Appendix A

### Appendix A.1. Physical Activity Package

File Content (JSON)

- Client ID {number}
- Activity level in a 10-min window in the period 1 March 2019 to 29 February 2020 {number}
- Activity level in a 10-min window in the period 1 March 2020 to 28 February 2021 {number}

The activity level of PA in a 10-min window shows how clients move in this time window. If the participant was medium active in each minute of the time window, then the value is 100. If he/she was medium active for 5 min in the time window, then the value is 50. For inactive times, the value is zero.

### Appendix A.2. Client Information Package

File Content (JSON)

- Client ID {number}
- Gender {text}
- Year of birth {number}
- Postcode of residence {number}
- Mobility Status {normal, low, with help, wheelchair}
- Housing Status {apartment, house}
- Height {number}
- Weight {number}

### Appendix A.3. Number of Population in the Cities

File Content (XLSX)

- District code {number}
- City code {number}
- Name of municipality {text}
- Population total, males, females {number}
- Average age total, males, females {number}

Czech statistics government published document: Population of municipalities of the Czech Republic [20].

### Appendix A.4. ZIP Code List

File Content (XLS)

- City name {text}
- ZIP code {number}
- Post name {text}
- District code {number}
- District name {text}

List of ZIP codes in parts of municipalities and municipalities without parts [19]. This document was published by the Czech post company.

## Appendix B

This appendix presents the statistical values for each hypothesis. Values are given for each pre-COVID (pre), during-COVID (during), big city, small city, living in a flat (flat) and living in a house (house) group compared.

**Table A1:** Statistical *p*-values for each hypothesis.

Hypothesis	*p*-Value	*p*-Value for the First Group Normality Test	*p*-Value of the Second Group Normality Test	Test Type
H01	0.0000407	0.0558 (pre)	0.4839 (during)	*t*-test
H02	0.0351	0.092 (pre)	0.2268 (during)	*t*-test
H03	0.000279	0.0246 (pre)	0.4113 (during)	Wilcox
H05	0.0003187	0.0029 (pre)	0.1223 (during)	Wilcox
H06	0.9216	0.0029 (Big city)	0.03804 (Small city)	Wilcox
H07	0.9981	0.1223 (Big city)	0.3249 (Small city)	*t*-test
H08	0.5802	0.0218 (flat)	0.132 (house)	Wilcox
H09	0.9982	0.008 (flat)	0.5 (house)	Wilcox
H10 (<80)	0.4713	0.3346 (pre)	0.5 (during)	*t*-test
H10 (80–85)	0.00137	0.0682 (pre)	0.0178 (during)	Wilcox
H10 (86–90)	0.0342	0.1599 (pre)	0.5 (during)	*t*-test
H10 (>90)	0.2342	0.0499 (pre)	0.5 (during)	Wilcox

**Table A2:** Statistical values for H04 for each month.

Month	*p*-Value	Normality Test Result Pre-COVID Group	Normality Test Result During-COVID Group	Test Type
January	0.5143	No	No	*t*-test
February	0.8778	No	No	*t*-test
March	0.0272	No	Yes	Wilcox
April	0.000166	No	No	Wilcox
May	0.000221	No	No	Wilcox
June	0.0014	No	No	*t*-test
July	0.0124	No	No	Wilcox
August	0.0051	No	No	Wilcox
September	0.1718	No	No	*t*-test
October	0.0683	No	No	Wilcox
November	0.0011	No	No	Wilcox
December	0.4095	No	No	Wilcox

**Table A3:** Statistical values (mean, std, median and interquartile) for relevant hypothesis. The values in the table are percentages. A value of 100% activity corresponds to PA in every minute of the active part of the day (from 7 am to 10 pm).

Hypothesis		Mean		Std		Median		Interquartile	
		Pre-COVID	During-COVID	Pre-COVID	During-COVID	Pre-COVID	During-COVID	Pre-COVID	During-COVID
H01		8.8944	7.8846	12.0414	11.4569	6.3407	5.097	12.8576	11.3085
H02		9.5937	8.4228	5.5547	5.0809	9.4685	7.3391	6.9067	6.7901
H03		14.0388	12.4687	13.8464	13.5239	11.6287	9.5267	10.2819	10.1811
H05		11.4504	10.33	6.4578	6.8865	10.3775	8.7298	9.0502	8.0299
H06 + H07	Big city	11.4504	10.33	6.4578	6.8865	10.3775	8.7298	10.3775	8.0299
	Small city	15.006	13.0793	17.2835	16.5888	11.0594	8.8027	11.1257	10.2064
H08 + H09	Flat	12.0011	10.5609	6.8931	7.1804	10.6546	8.6143	10.2723	9.6763
	House	11.0172	9.6469	5.8998	5.3178	10.8687	9.9616	5.7273	7.0575
H10 (<80)		12.21112	10.8139	16.7812	14.0844	8.4119	7.3049	7.9115	8.5652
H10 (80–85)		11.9232	9.6699	6.8988	6.5215	11.0594	8.7298	9.3082	8.0688
H10 (86–90)		15.1559	14.5726	15.2016	16.6061	12.7166	9.7711	8.6855	11.1537
H10 (>90)		11.6059	10.2137	6.6508	7.5455	10.4028	8.3309	9.9492	9.4134

**Table A4:** Questions for the correlation of BMI with the level of physical activity.

Hypothesis	R-Value
H11	0.004
H12	0.121
